# Caroli disease incidentally discovered in a 23-years old male: A case report

**DOI:** 10.1016/j.radcr.2024.09.080

**Published:** 2024-10-04

**Authors:** Luis F. Bonilla Larrama, Cesar U. Alas-Pineda, Anahi G. Pacheco, Victoria S. Díaz-Cerrato, Karla P. Molinero Leiva

**Affiliations:** a Centro Médico Quirúrgico CIMEQ, El Progreso, Yoro, Honduras; b Departamento de Medicina Interna. Hospital Nacional Dr. Mario Catarino Rivas, San Pedro Sula, Cortes, Honduras; c Universidad Católica de Honduras, Campus San Pedro y San Pablo, Facultad de Medicina y Cirugía, San Pedro Sula, Honduras; d Escuela Universitaria de Ciencias de la Salud, Universidad Nacional Autónoma de Honduras en el Valle de Sula, San Pedro Sula, Honduras

**Keywords:** Caroli disease, Magnetic resonance cholangiopancreatography, Intrahepatic bile duct dilation, Case report

## Abstract

This case report describes a 23-year-old male who was incidentally diagnosed with Caroli disease while investigating symptoms of recurrent upper quadrant pain and fever. Imagining studies, including magnetic resonance cholangiography revealed the “central dot sign,” which is pathognomonic for this condition, along with cystic dilatations of the intrahepatic bile ducts and the presence of biliary stones. The diagnosis was confirmed and treatment with ursodeoxycholic acid led to a notable reduction in symptoms. This case underscores the need for further research and understanding to develop more effective diagnostic and treatment strategies. Regular follow-up remains essential for managing disease progression and preventing complications.

## Introduction

Caroli disease is a rare genetic condition, known as cavernous ectasia of the bile ducts. It causes segmental dilation of the major intrahepatic duct, which appears cystic on imaging and histopathological examination [1]. It is part of the clinical spectrum of autosomal recessive polycystic kidney disease (ARPKD), resulting from mutations in the PKHD1 gene [2]. These mutations affect the fibrocystin protein expressed in pancreatic cells, liver cholangiocytes, and renal tubular cells, leading to polycystic kidney and liver disease [1]. The exact cause of the disease remains unknown, and it was first described by Jacques Caroli in 1958 [3].

## Case presentation

We present the case of a 23-year-old male from a rural area in Honduras, who works as a dairy farmer. The patient reported a history of intermittent right upper quadrant pain since the age of 13. This pain was sporadic, with periods of remission, and had been managed with oral analgesics and antispasmodics, such as lysine clonixinate and Propinox hydrochloride. Recently, the pain had significantly increased in intensity and became persistent, prompting the patient to seek care at a tertiary care facility.

**Fig. 1:**
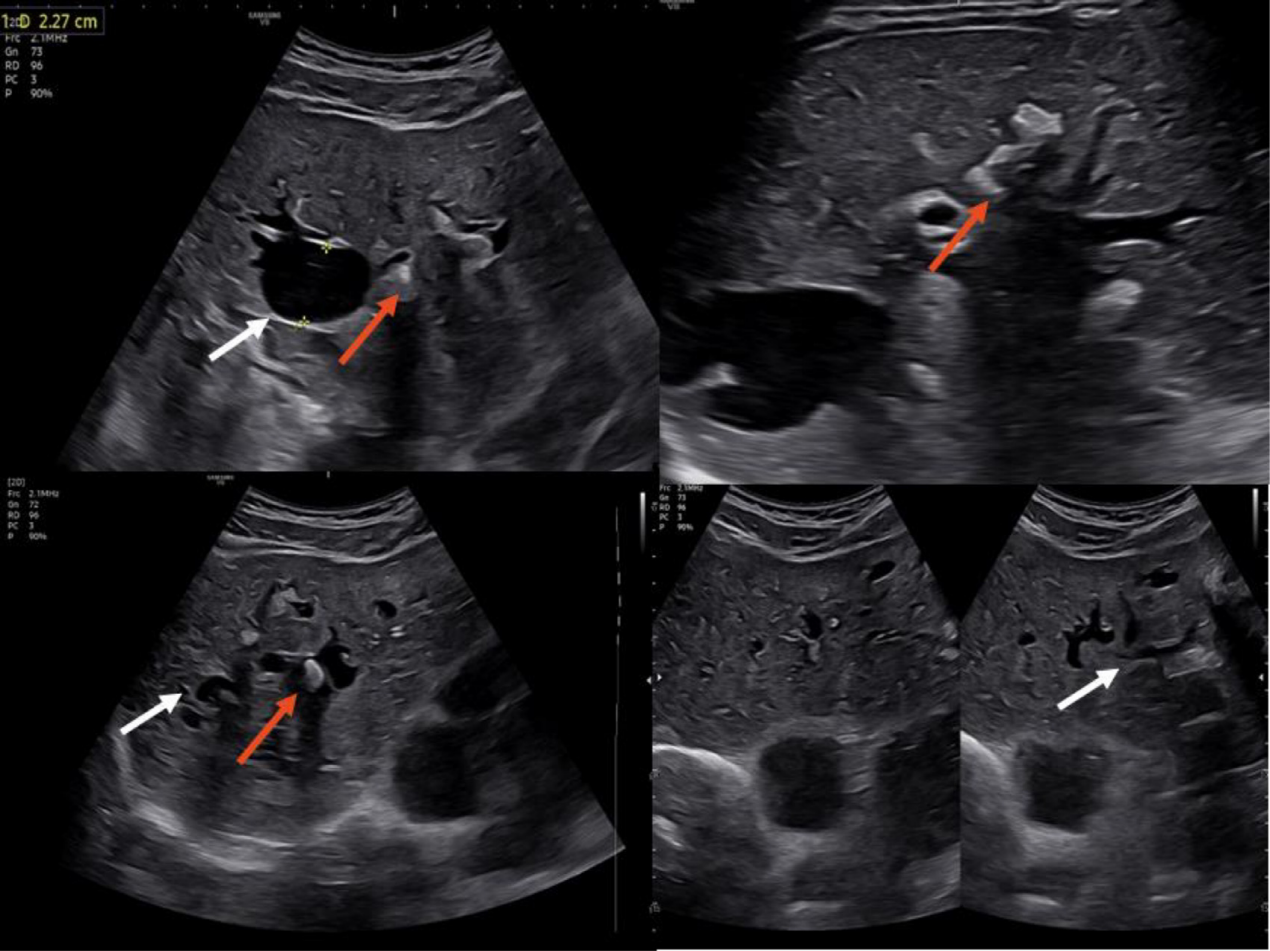
Abdominal ultrasound showing cystic dilation of the intrahepatic bile ducts (white arrows), with multiple stones inside (red arrows).

On physical examination, there were no signs of hepatomegaly, a positive McBurney's sign, jaundice, fever, or other associated symptoms. Upon admission, intravenous analgesia was administered, and additional diagnostic tests and an abdominal ultrasound were ordered. Initial laboratory tests showed a total bilirubin level of (0.8 mg/dL) and a direct bilirubin level of (0.1 mg/dL). Alkaline phosphatase at 124 U/L, while alanine aminotransferase (ALT) and aspartate aminotransferase (AST) were at 16 U/L and 19 U/L, respectively. The complete blood count revealed hemoglobin of 14.60 g/dL, hematocrit of 45.40%, white blood cells of 7.45 /uL, platelets of 299/uL, and neutrophils at 58.10%. Other hematological and biochemical parameters were within normal limits. The abdominal ultrasound revealed sacular dilation of the intrahepatic bile ducts in both lobes, associated with multiple hyperechoic foci suggestive of intrahepatic stones (Fig. 1).

No stones were identified within the gallbladder and the extrahepatic bile ducts could not be adequately assessed. In light of these findings and the suspicion of biliary pathology, a magnetic resonance cholangiography (MRCP) was requested.

**Fig. 2:**
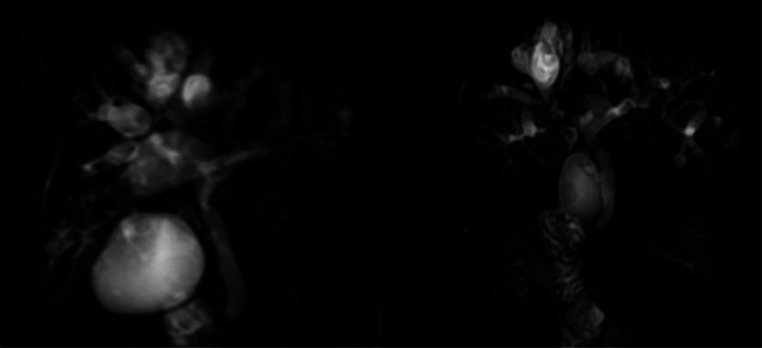
The MRCP image shows multiple areas of cystic and fusiform dilations of the peripheral intrahepatic bile ducts. The cystic dilations are primarily peripheral and larger at the periphery than in the center.

The MRCP revealed saccular and fusiform, multifocal, and peripheral dilation of the intrahepatic bile ducts, with diameters ranging from 0.5 cm to 2.8 cm (Fig. 2).

**Fig. 3:**
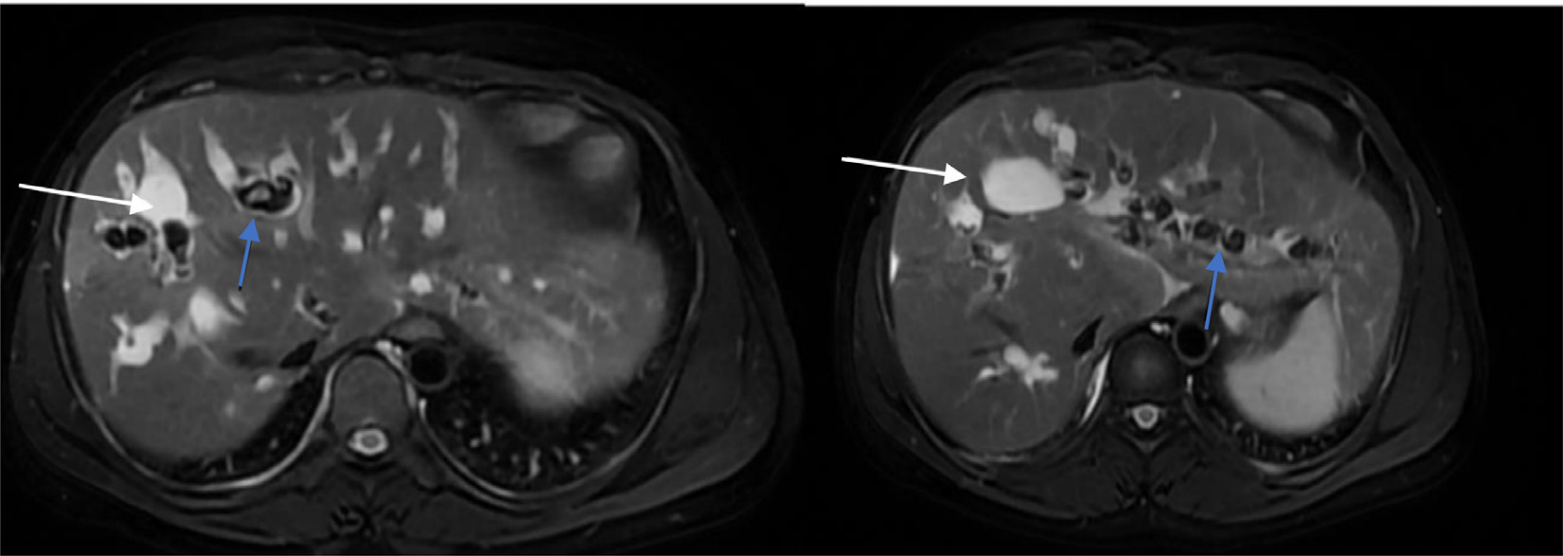
The axial T2-weighted MRI image shows multiple multifocal saccular cystic dilations of the intrahepatic bile ducts (white arrows) containing numerous stones (blue arrows).

Numerous stones were identified within these ducts, ranging in size from 4 mm to 16.2 mm (Fig. 3).

**Fig. 4:**
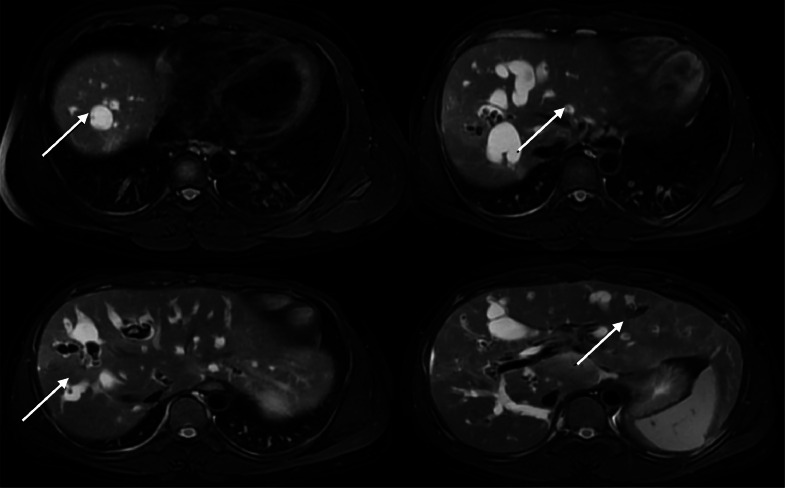
“Central dot sign” is indicated by the arrows. (white arrows).

A slight dilation of the extrahepatic bile duct was also observed. However, the gallbladder did not contain any stones. Additionally, the pathognomonic “central dot sign” was observed (Fig. 4).

These imaging findings are indicative of Caroli disease, convoluted by the presence of multiple intrahepatic stones.

Based on the clinical, laboratory, and imaging findings, the patient was diagnosed with Caroli disease. Treatment with ursodeoxycholic acid was initiated to improve bile flow and prevent the formation of additional biliary stones. During the 3-month follow-up, the patient demonstrated significant improvement in symptoms, and the abdominal pain resolved. Regular follow-up will continue through laboratory tests and imaging studies, which are essential to adjust the treatment according to the progression of the disease, ensuring the patient's well-being and detecting potential complications.

## Discussion

Caroli disease is a rare congenital condition characterized by segmental and multifocal dilation of the intrahepatic bile ducts. When this condition occurs without hepatic fibrosis, it is referred to as Caroli disease; whereas when associated with congenital hepatic fibrosis, it is known as Caroli syndrome [4]. Classified as Type V in the Todani classification system [5], this pathology has an extremely low prevalence, affecting fewer than 1 in a million individuals, with a higher incidence in females at a ratio of 1.8:1 compared to males [6].

This condition presents as a congenital malformation of the intrahepatic duct, characterized by segmental cystic dilations of the intrahepatic ducts [7], its potential mechanisms include obstruction of the hepatic artery leading to bile duct ischemia and cystic dilation, excessive growth of the bile duct epithelium and connective tissue development, and failure of normal remodeling of the ductal plates at the portal ducts [6]. Additionally, it is associated with an increased incidence of biliary stones, cholangitis, and hepatic abscesses, while cirrhosis and portal hypertension are absent, and an association with renal tubular ectasia or similar cystic renal diseases [7].

Symptoms of Caroli disease can vary significantly among patients and often overlap with other hepatic and biliary conditions [1]. Typical symptoms include recurrent abdominal pain in the right upper quadrant, fever, jaundice, and, in some cases, hepatomegaly [7]. Due to the variable nature of the symptoms, diagnosis can be challenging and often requires a combination of imaging studies and laboratory tests [1]. The clinical course may be asymptomatic in the first 5-20 years or symptoms may present infrequently over the patient's lifetime [7]. Imaging studies are essential for diagnosis, with magnetic resonance cholangiopancreatography (MRCP) being the most useful tool [8]. The most pathognomonic radiological feature of this disease is the “central dot sign,” representing the portal vessels contrasted with the cystic dilations of the bile ducts [5].

In the presented case, a 23-year-old male with a history of recurrent abdominal pain in the right upper quadrant since age 13 showed a recent increase in pain intensity accompanied by fever. These symptoms led to an ultrasound and MRCP, which revealed the “central dot sign,” thus confirming the diagnosis of Caroli disease. The imaging showed saccular dilations of the intrahepatic bile ducts, the presence of stones, and a slight dilation of the extrahepatic bile duct, all indicative of this disease [9].

Differential diagnosis for Caroli disease includes a range of hepatic and biliary pathologies such as primary sclerosing cholangitis, recurrent pyogenic cholangitis, polycystic liver disease, choledochal cysts, biliary papillomatosis, and Von Meyenburg complex [6]. These conditions share similar symptoms and can complicate diagnosis, highlighting the importance of a thorough and multidisciplinary diagnostic approach.

Management of Caroli's disease focuses on symptom treatment and complication prevention. Patients typically receive antibiotics to manage episodes of cholangitis and ursodeoxycholic acid to improve bile flow and prevent the formation of additional biliary stones. Given the impact on the hepatobiliary system, regular follow-up is crucial, including abdominal ultrasounds, monitoring of ALT and AST levels, and periodic blood counts [10].

## Conclusion

The case involves a patient with Caroli disease, a condition marked by saccular dilation of the intrahepatic bile ducts and the pathognomonic central dot sign. Diagnosis was based on clinical presentation, imaging studies, and family history. Management of Caroli disease is individualized, depending on the extent of intrahepatic involvement. In this case, the patient is monitored regularly with laboratory tests and imaging to detect complications and track disease progression.

## Author contributions

Guarantor of integrity of the entire study: Cesar A. Pineda; Study concepts and design: Cesar A. Pineda and Anahi G. Pacheco; Literature research: Anahi G. Pacheco; Clinical studies: Cesar A. Pineda and Luis Bonilla; Data Collection: Victoria Diaz Cerrato; Manuscript preparation: Anahi G. Pacheco and Karla Molinero; Manuscript editing: Anahi G. Pacheco and Karla Molinero.

## Patient consent

We, hereby declare that informed consent has been obtained from the patient referenced in this publication. The patient has been fully informed about the nature and purpose of the publication.

## Notes

This case report was prepared following the CARE Guidelines [11].
